# County-level spatiotemporal distribution of fluoroquinolone-resistant Enterobacteriaceae in outpatient settings of the Veterans’ Health Administration, 2000–2017

**DOI:** 10.1017/ice.2022.291

**Published:** 2023-09

**Authors:** Matthew W. Smith, Margaret Carrel, Michihiko Goto

**Affiliations:** 1 Center for Access & Delivery Research and Evaluation (CADRE), Iowa City VA Health Care System, Iowa City, Iowa; 2 Division of Infectious Diseases, Department of Internal Medicine, University of Iowa Carver College of Medicine, Iowa City, Iowa; 3 Department of Geographical and Sustainability Sciences, College of Liberal Arts and Sciences, University of Iowa, Iowa City, Iowa

## Abstract

Fluoroquinolone resistance among Enterobacteriaceae is a notable challenge for appropriate empiric therapy in outpatient settings. We describe the spatial distribution of fluoroquinolone resistance and its chronological change between 2000 and 2017 in the nationwide Veterans’ Health Administration system. We found spatially concentrated increasing prevalence in the 2000s, followed by spatial dispersion in the 2010s.

With broad-spectrum activity and excellent oral bioavailability, fluoroquinolones enable clinicians to treat serious bacterial infections in the outpatient setting. However, since their development in the 1970s, bacterial resistance to fluoroquinolones has steadily increased.^1^ Enterobacteriaceae are responsible for a large burden of disease, accounting for >20% of all healthcare-associated infections.^2^ Fluoroquinolone resistance among Enterobacteriaceae rose from 0%–2% to 10%–12% from 1990 to 2000,^3,4^ and this trend continued during the following decade.^5^

Most of the data regarding fluoroquinolone resistance among Enterobacteriaceae are descriptive in nature, primarily coming in the form of sentinel surveillance data or regional data.^6^ In the United States, there is a dearth of information characterizing resistance patterns in space and time from population-level data with wider geographic coverage to improve our understanding of the spread of resistance over time. The European Centre for Disease Prevention and Control (ECDC) publishes data annually for the prevalence of antimicrobial resistance in member nations.^7^ The trends elucidated in the ECDC reports suggest that geographic variabilities in antimicrobial resistance prevalence exist at smaller scales than are currently tracked and reported in the United States.

The Veterans’ Health Administration (VHA) is the only integrated healthcare system in the United States, serving patients in all states and territories. In this study, we have described county-level geographic variation in fluoroquinolone resistance among Enterobacteriaceae from 2000 to 2017 and its change over time.

## Methods

This study was approved by the institutional review board of the University of Iowa. Informed consent was waived for this retrospective cohort study.

### Population

We analyzed a retrospective cohort of patients who received care at outpatient settings within the VHA in the United States with positive clinical culture specimens for Enterobacteriaceae from January 1, 2000, through December 31, 2017. We included all patients with at least 1 positive culture for Enterobacteriaceae from any clinical specimen. The study population included residents from the 3,108 counties in the 48 continental states and the District of Columbia who received care at 1,253 healthcare facilities (168 hospitals and 1,085 outpatient clinics).

### Data and variables

We extracted data from the Corporate Data Warehouse of the VHA. Isolates were classified as resistant if their susceptibilities were reported as either intermediate or resistant to any fluoroquinolone (ie, ciprofloxacin, ofloxacin, levofloxacin, gatifloxacin, or moxifloxacin). No facility had adopted a lowered breakpoint for fluoroquinolones, which was published by the Clinical and Laboratory Standards Institute (CLSI) in 2019. No information was available on bacterial genetics or mechanisms of resistance. Culture specimens collected in institutionalized settings (ie, cultures acquired >48 hours after admission to acute care, long-term care, and mental health units) were excluded. All isolates were assigned to counties based on geocoded residential addresses of patients.

### Measurements

The primary outcome measure was the county-level fluoroquinolone resistance rate, calculated as the proportion of number of unique patients with at least 1 positive fluoroquinolone-resistant Enterobacteriaceae clinical culture divided by the total number of unique patients with at least 1 positive Enterobacteriaceae culture in the county for each calendar year. For spatial analyses, we aggregated data to terciles (2000–2005, 2006–2011, 2012–2017) to estimate county-level resistance rates.

### Analysis

First, we summarized the overall fluoroquinolone resistance rate for each year with descriptive statistics. Next, we summarized county-level resistance rates for each tercile and assigned them to the spatial centroids of counties. We then estimated and mapped the proportion of resistant isolates using the empirical Bayes smoothing technique^8^ which computed a weighted average between the raw rate for each county and the nationwide average, with weights proportional to the sample size at each county. This approach allowed us to avoid the uncertainty inherent in rates for counties with a very small number of isolates. Lastly, we conducted a geospatial cluster analysis on the estimated county-level fluoroquinolone-resistance rates to detect any spatial clustering in resistance using discrete Poisson modeling. We assumed circular clustering, and areas with relative risk >1.4 were compared to the surrounding area and detected as clusters with α = 0.05. We used GeoDa version 1.12 software for empirical Bayes estimation and SaTScan version 0.3.92 software for cluster analysis.

## Results

Of the 107,000,000 patient years of care that occurred in the VHA from 2000 to 2017, 1,621,762 patient years included at least 1 positive culture for Enterobacteriaceae, with 2,545,430 unique culture specimens. The final cohort included 1,980,065 specimens after the exclusion, and antimicrobial susceptibility data for fluoroquinolones were available for 91% of cases.

**Fig. 1.**
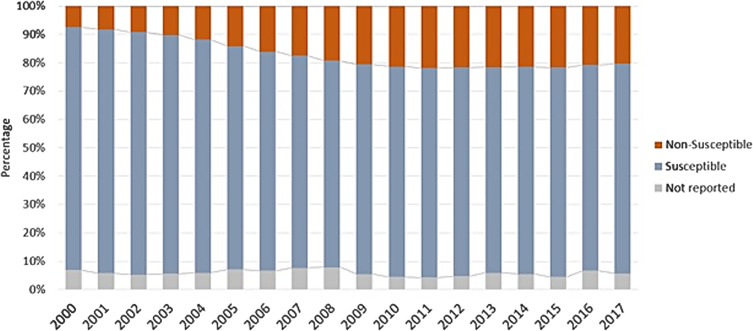
Overall fluoroquinolone resistance rates among Enterobacteriaceae by year.

**Fig. 2.**
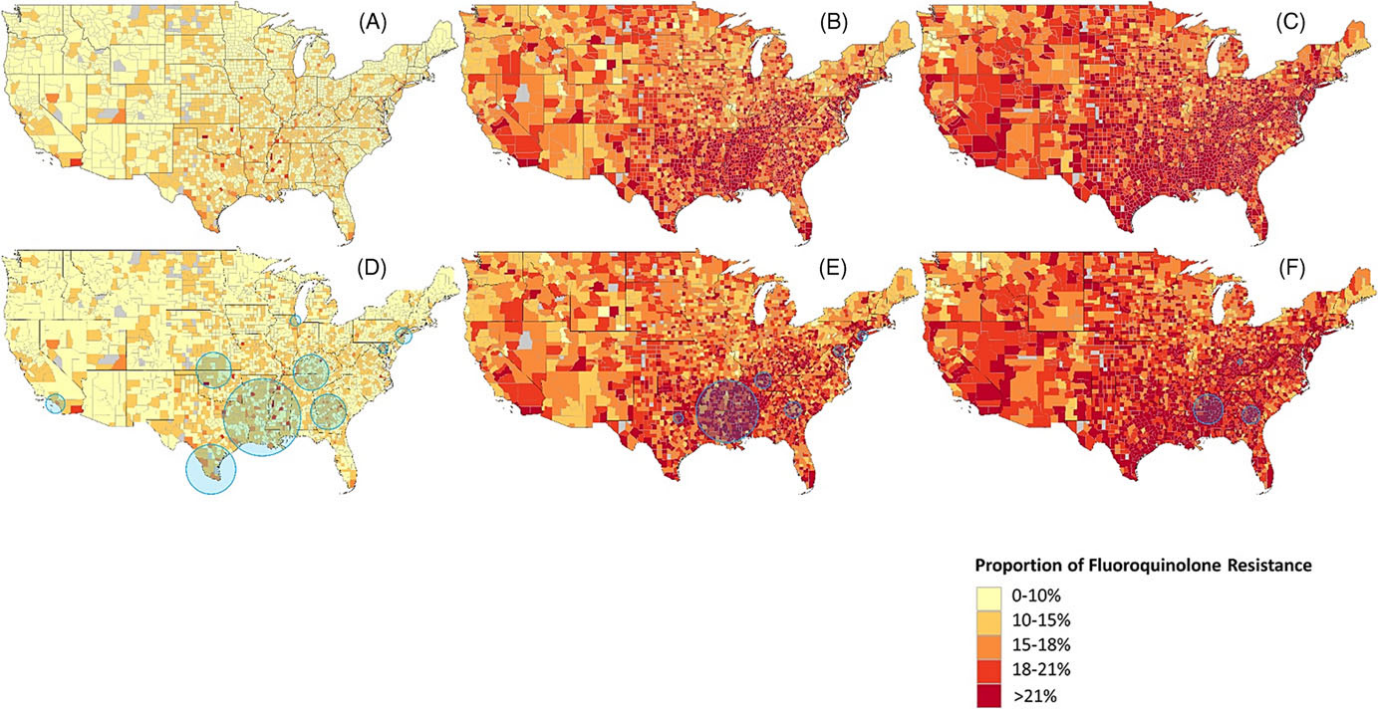
Proportion of fluoroquinolone-resistant Enterobacteriaceae by county. Top row: (A) incidence during 2000–2005, (B) 2006–2011, and (C) 2012–2017. Bottom row: (D) incidence with cluster analysis overlay during 2000–2005, (E) 2006–2011, and (F) 2012–2017.

The fluoroquinolone resistance rate among all Enterobacteriaceae (Fig. 1) was 8% in the year 2000. Resistance peaked at 22% in 2011, and this rate plateaued during 2011–2017. During 2000–2005 (Fig. 2A), the nationwide rate of fluoroquinolone resistance was low, with most counties having 0%–10% resistance, excepting a few outlier counties in the South region and southern California. During 2006–2011 (Fig. 2B) resistance increased, with many counties having resistance rates of 15%–21%, and large portions of the South region, southern California, and the Northeast region having rates >21%. Finally, during 2012–2017 (Fig. 2C), fluoroquinolone resistance became widespread, with most counties having >21% resistance.

We used cluster analysis to detect outlier regions of high fluoroquinolone resistance. The analysis from 2000 to 2005 (Fig. 2D) identified regions with increased risk of fluoroquinolone-resistant Enterobacteriaceae, including areas not readily apparent in the descriptive maps, such as the Northeast, southern Texas, and northern Illinois. Similar findings are seen during 2006–2011 (Fig. 2E), with an increased risk of resistance identified in the southeastern states, Texas, and the eastern coast. Three small clusters were identified in the Southeast region during 2012–2017 (Fig. 2F).

## Discussion

Mapping resistance over space and time revealed distinct regional variability in the early 2000s, with clusters demonstrating areas of nascent fluoroquinolone resistance in Enterobacteriaceae in the Northeast region, Texas, and northern Illinois. Over time, this resistance appeared to expand outward from these clusters with eventual homogenous, high-level resistance across the country, indicating geographic dispersion of resistance from highly concentrated areas to low-prevalence areas. Future studies can assess the county-level factors associated with the earlier emergence of fluoroquinolone resistance and mechanisms of spatial dispersion over time.

This analysis benefitted from the utilization of data from the VHA health system, which enabled us to obtain patient-level microbiologic data and residential addresses and allowed us to describe the prevalence in greater detail using clustering analysis. In comparison, data at the state and national levels did not allow us to detect highly concentrated areas or to conduct spatial analysis,^9^ and data at the level of individual hospitals (ie, antibiograms) typically do not consider where patients reside.

The generalizability of findings is limited by VHA patients, who are predominantly older and male compared to the US population. Additionally, 9% of clinical isolates did not have susceptibility reports, raising the possibility that nuances were missed in the analysis, and we did not have information about facility-level laboratory susceptibility testing methods, meaning that susceptibility data may have varied between facilities. Finally, no patient-level or county-level variables were considered for adjustment. The ability to further stratify data could allow for the exploration of additional relevant geographic trends, which we were not able to assess.

Fluoroquinolone resistance among Enterobacteriaceae showed spatial heterogeneity with clustering during the early 2000s. Over the following decade, geographical dispersion of resistance occurred, and resistance rates peaked and then plateaued from 2011 to 2017. Prospectively tracking resistance via the use of geospatial analysis can facilitate the early discovery of resistance pockets and has the potential to disseminate information about emerging pathogens.

## Supplementary material

For supplementary material accompanying this paper visit https://doi.org/10.1017/ice.2022.291. click here to view supplementary material
