# Supplementary figures and images for: County-level spatiotemporal distribution of fluoroquinolone-resistant Enterobacteriaceae in outpatient settings of the Veterans’ Health Administration, 2000–2017
Source: Infect Control Hosp Epidemiol. 2022 Dec 2;44(9):1497–9. doi: 10.1017/ice.2022.291 (PMC10507512; doi:10.1017/ice.2022.291)

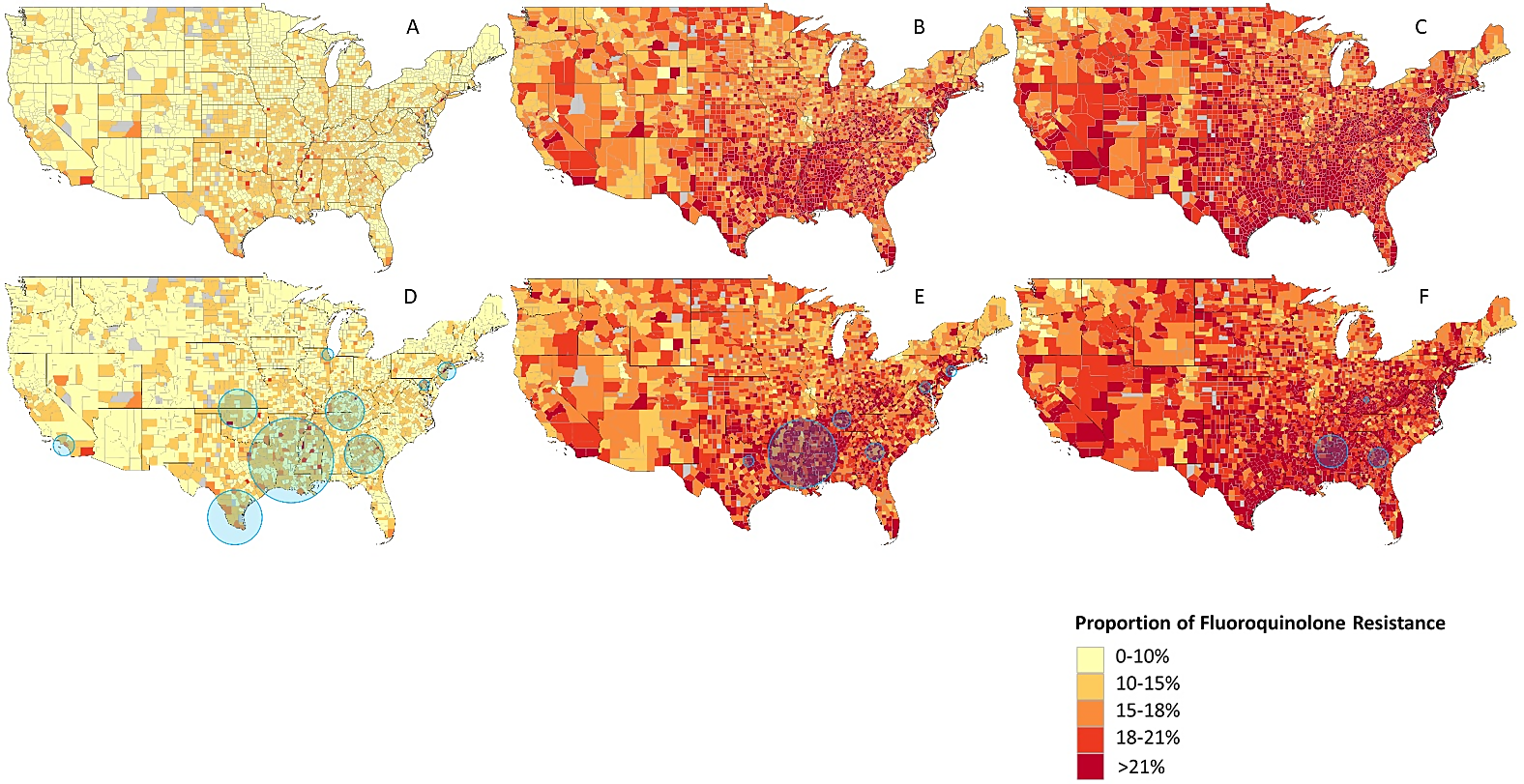

Supplement: Supplementary file 1 [file S0899823X22002914sup.zip › S0899823X22002914sup001.tiff]

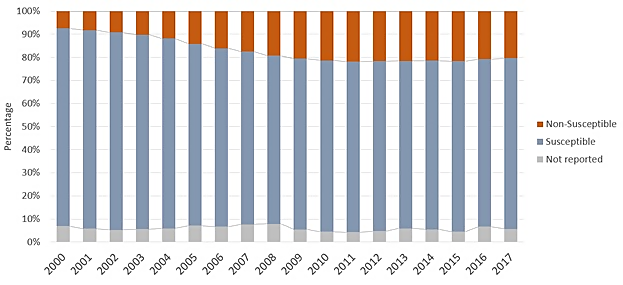

Supplement: Supplementary file 1 [file S0899823X22002914sup.zip › S0899823X22002914sup002.tiff]
